# Long Non-coding RNA LincRNA-EPS Inhibits Host Defense Against *Listeria monocytogenes* Infection

**DOI:** 10.3389/fcimb.2019.00481

**Published:** 2020-01-22

**Authors:** Federica Agliano, Katherine A. Fitzgerald, Anthony T. Vella, Vijay A. Rathinam, Andrei E. Medvedev

**Affiliations:** ^1^Department of Immunology, UConn Health School of Medicine, Farmington, CT, United States; ^2^Program in Innate Immunity, Division of Infectious Diseases and Immunology, Department of Medicine, University of Massachusetts Medical School, Worcester, MA, United States

**Keywords:** lncRNAs, infection, *Listeria monocytogenes*, innate immunity, lincRNA-EPS

## Abstract

Long non-coding RNAs (lncRNAs) have emerged as key regulators of gene expression in several biological systems. The long intergenic RNA-erythroid pro-survival (lincRNA-EPS) has been shown to play a critical role in restraining inflammatory gene expression. However, the function of lincRNA-EPS during bacterial infections remains unknown. Here, we demonstrate that following infection with the intracellular bacterium *Listeria monocytogenes*, both mouse macrophages and dendritic cells lacking lincRNA-EPS exhibit an enhanced expression of proinflammatory cytokine genes, as well as an increased expression of the inducible nitric oxide synthase (*iNos)* and nitric oxide (NO) production. Importantly, we found that lincRNA-EPS^−/−^ mice intraperitoneally infected with *L. monocytogenes* exhibit lower bacterial burdens in spleen and liver and produce more NO than control mice. Furthermore, lincRNA-EPS^−/−^ mice are less susceptible to a lethal dose of *L. monocytogenes* than wild type (WT) mice. Collectively these findings show that lincRNA-EPS suppresses host protective NO expression and impairs the host defense against *L. monocytogenes* infection.

## Introduction

Long non-coding RNAs (lncRNAs) have recently emerged as key regulators of gene expression, modulating a variety of transcriptional and translational events (Lee, 2012; Morris and Mattick, 2014). LncRNAs are arbitrarily defined as non-coding RNAs longer than 200 nucleotides, a feature that distinguishes them from other non-coding transcripts, such as micro RNAs (miRNAs) and small nucleolar RNAs (snoRNAs). Based on their genomic location lncRNAs are classified in long intergenic non-coding RNAs (lincRNAs), bidirectional lncRNAs, intronic non-coding RNAs, and natural antisense transcripts (NATs) (Murphy and Medvedev, 2016). The role of lncRNAs in host defense is just emerging. LncRNA-ACOD1 was shown to facilitate viral infections by directly binding and enhancing the catalytic activity of the metabolic enzyme glutamic-oxaloacetic transaminase (GOT2) (Wang et al., 2017). During *Salmonella enterica Typhimurium* infection, lncRNA NEAT1 was demonstrated promote the expression of immune related genes and blocking Salmonella replication (Imamura et al., 2018).

LincRNA-EPS was discovered in 2011 as a mammalian lincRNA involved in erythroid differentiation (Hu et al., 2011). Recently, lincRNA-EPS was shown to control nucleosome positioning and the transcription of immune-related genes (IRGs) (Atianand et al., 2016). LincRNA-EPS is abundantly expressed and its expression is downregulated in response to microbial ligands including lipopolysaccharide (LPS) (Atianand et al., 2016). However, whether lincRNA-EPS plays a role during bacterial infections is not known. *L. monocytogenes* is a gram-positive, facultative intracellular pathogen and is the causative agent of foodborne listeriosis. In immunocompromised individuals *L. monocytogenes* can lead to gastritis, septicemia and meningitis (Gellin and Broome, 1989; Mehmood et al., 2017); whereas, in pregnant women this pathogen can cause infection to the fetus and septic abortion (Pamer, 2004).

Here, we show the role of lincRNA-EPS in host defense utilizing *L. monocytogenes* as a model pathogen. *L. monocytogenes* infection induces the downregulation of lincRNA-EPS expression consistent with the previous report. Macrophages from lincRNA-EPS^−/−^ mice express significantly higher levels of proinflammatory cytokines, such as TNF-α, IL-6, and CCL5 than control cells in response to *L. monocytogenes* infection. Similarly, lncRNA-EPS^−/−^ dendritic cells exhibited increased TNF-α and IL-6 expression. Importantly, lincRNA-EPS^−/−^ mice controlled *L. monocytogenes* infection and survived significantly better than wild type mice. Taken together, these results demonstrate that during *L. monocytogenes* infection, lincRNA-EPS suppresses bacterial clearance and host defense responses.

## Materials and Methods

### Mice and Primary Cells Isolation

C57BL/6J mice were from the Jackson Laboratory (BarHarbor, ME). LincRNA-EPS^−/−^mice were provided by Dr. Katherine Fitzgerald (University of Massachusetts Medical School, Worcester, MA). Mice were housed in the University of Connecticut Health Center animal facility, and 6–16 week-old male and female mice were used. To generate bone marrow derived macrophages (BMDMs), bone marrow cells from femurs and tibias were differentiated for 7 days in DMEM (Gibco, Dublin, Ireland), supplemented with 10% FBS (Atlanta Biologicals, Flowery Branch, GA), 2 mM L-glutamine, 100 U/ml penicillin, 100 μg/ml streptomycin, and 15% L929 cell-conditioned, macrophage-colony-stimulating factor-containing supernatant. Bone marrow derived dendritic cells (BMDCs) were generated by differentiating bone marrow cells in RPMI 1640 medium supplemented with 10% FBS, 100 U/ml penicillin, 100 μg/ml streptomycin, 50 μM 2-mercaptoethanol, and recombinant GM-CSF (20 ng/ml) for 10 days. All animal procedures and experiments were approved by the Institutional Animal Care and Use Committee (IACUC), the ethics committee responsible for reviewing the use of animals in research and teaching at the University of Connecticut.

### *Listeria monocytogenes* Culture and Infection

*L. monocytogenes* (10403S) was provided by Dr. Kamal Khanna (New York University School of Medicine, New York, NY). *L. monocytogenes* was cultured in brain heart infusion (BHI) media containing 10 μg/ml chloramphenicol. Bacteria were grown to mid log phase and washed in PBS. For *in vitro* experiments bacteria were added to cells cultured in antibiotic-free medium at a multiplicity of infection (MOI) of 1 or 10 for 1.5 h. Cells were washed in PBS and incubated in complete medium supplemented with 50 μg/ml gentamycin for the indicated time points. For *in vivo* experiments, mice were i.p. administered *L. monocytogenes* 10^5^ CFU. At the selected time point, spleens, and livers were homogenized in PBS containing protease and phosphatase inhibitors (Thermo Fisher Scientific, Waltham, MA) for further analysis. For survival experiments mice were i.p. administered *L. monocytogenes* 5 × 10^5^ CFU and observed for 10 days after infection.

### Bacterial CFUs Count

BMDMs and BMDCs were grown overnight in 6-well plates (2 × 10^6^ cells/well) or in 24-well plates (0.4 × 10^6^ cells/well), infected for 1.5 h with *L. monocytogenes* (MOI of 1) in antibiotic free medium, washed with PBS, incubated in completed medium containing 50 μg/ml gentamicin for the indicated times and lysed in PBS containing 1% Triton X-100. Spleens and livers from *L. monocytogenes*-infected mice were homogenized in PBS containing protease and phosphatase inhibitors (ThermoFisher Scientific). The number of CFUs per well or per gram of organ homogenates was determined by plating 10-fold serial dilutions of lysates on BHI agar plates containing 10 μg/ml chloramphenicol. Plates were incubated at 37°C and CFUs were counted after 18–24 h.

### Extraction of RNA and Quantitative Real Time PCR

Total RNA was purified from cells and tissues using RNeasy RNA extraction kit (Qiagen) as per the manufacturer's instructions. Genomic DNA was eliminated using on-column DNase I digestion (Qiagen) and NanoDrop 2000 was used in order to measure RNA quality. cDNA was prepared from equal amounts of total RNA (50–1,000 ng), using iScript cDNA synthesis kit (Bio-Rad). The following primers were used—*Gapdh*: 5′-GCTGACCTGCTGGATTACATT-3′ (forward), 5′-GTTGAGAGATCATCTCCACCA-3′ (reverse); *Tnfa*: 5′-CCCAGGCAGTCAGATCATCTTC-3′ (forward), 5′-GCTTGAGGGTTTGCTACAACATG-3′ (reverse); *iNos*: 5′-CGCCTTCAACACCAAGGTTG-3′ (forward), 5′TGGGGACAGTCTCCATTCCCA-3′; *Il6*: 5′-TCAGGAAATTTGCCTATTGAAAATTT-3′ (forward), 5′-GCTTTGTCTTTCTTGTTATCTTTTAAGTTGT-3′ (reverse); *Ccl5*: 5′-GAGTGACAAACACGACTGCAAGAT-3′ (forward), 5′-CTGCTTTGCCTACCTCTCCC T-3′ (reverse); *Kc*: 5′-CCATGGCTGGGATTCACC-3′ (forward), 5′GACCATTCTTGAGTGTGGCTATGAC-3′ (reverse); 16S V6: 5′-TCGATGCAACGCGAAGAA-3′-(forward), 5′-ACATTTCACAACACGAGCTGACGA-3′ (reverse). Gene expression levels were normalized to *Gapdh* as housekeeping gene. Relative mRNA expressions were calculated according to the 2^−ΔΔ*t*^ calculation method. Specificity of PCR amplifications was assessed by melting curve analysis.

### Cytokine Secretion and NO Release

Supernatants from cell cultures and tissue homogenates were cleared by centrifugation (2,000 g, 10 min), and cytokine levels were determined by ELISA using commercial kits: Ccl5/Rantes (R&D Systems), Cxcl1/KC (R&D Systems), IL-6 (BioLegend, San Diego, CA), TNF-α (BioLegend, San Diego, CA), IL-1β (BioLegend, San Diego, CA). Nitrite (NO_2−_) concentrations in the supernatants and organ homogenates were examined by the Griess method. In brief, 1% sulfanilamide in 2.5% phosphoric acid-0.1% *n*-1-napthylethylenediamide dichlorique was added to 50 μl of samples and after 20 min of incubation absorbance was read at 540 nm. NO_2−_ was quantified using sodium nitrite (NaNO_2_) as a standard.

### Western Blot Analysis

BMDMs were infected with *L. monocytogenes* as described above and lysed in lysis buffer (50 mM Tris–HCl, pH 7.4, 150 mM NaCl, 1% Triton X-100, 1 mM EDTA, 5 mM NaF, 2 mM sodium orthovanadate, 1 mM PMSF, 1X complete protease inhibitors). The whole cell lysates were mixed with Laemmli buffer (50 mm Tris–Cl, pH 6.8, 10% glycerol, 2% SDS, 0.1% bromophenol blue, 5% 2-mercaptoethanol), boiled for 5 min, separated on 4–20% polyacrylamide gels (BioRad), transferred to nitrocellulose membranes (Bio-Rad), blocked, and probed with the following primary antibodies: monoclonal Ab to iNOS (clone D6B6S, Cell Signaling) and polyclonal Ab to β-actin (Sigma). Membranes were developed using an ECL plus chemiluminescence kit (Thermo Fisher Scientific) and protein quantification on the Western blots was performed using GeneTools software (Syngene).

### Statistics

The statistical significance was determined with unpaired, two-tailed Student's *t*-test and the survival data were analyzed using Log-rank (Mantel-Cox) test. *p* ≤ 0.05 was considered statistically significant. Data were processed by the GraphPad Prism 8.0 software package (Graph Pad Software, San Diego, CA) and expressed as mean ± SEM of at least two independent experiments.

## Results

### LincRNA-EPS Restrains *L. monocytogenes*-Induced Immune Gene Expression in Mouse Macrophages

**Figure 1:**
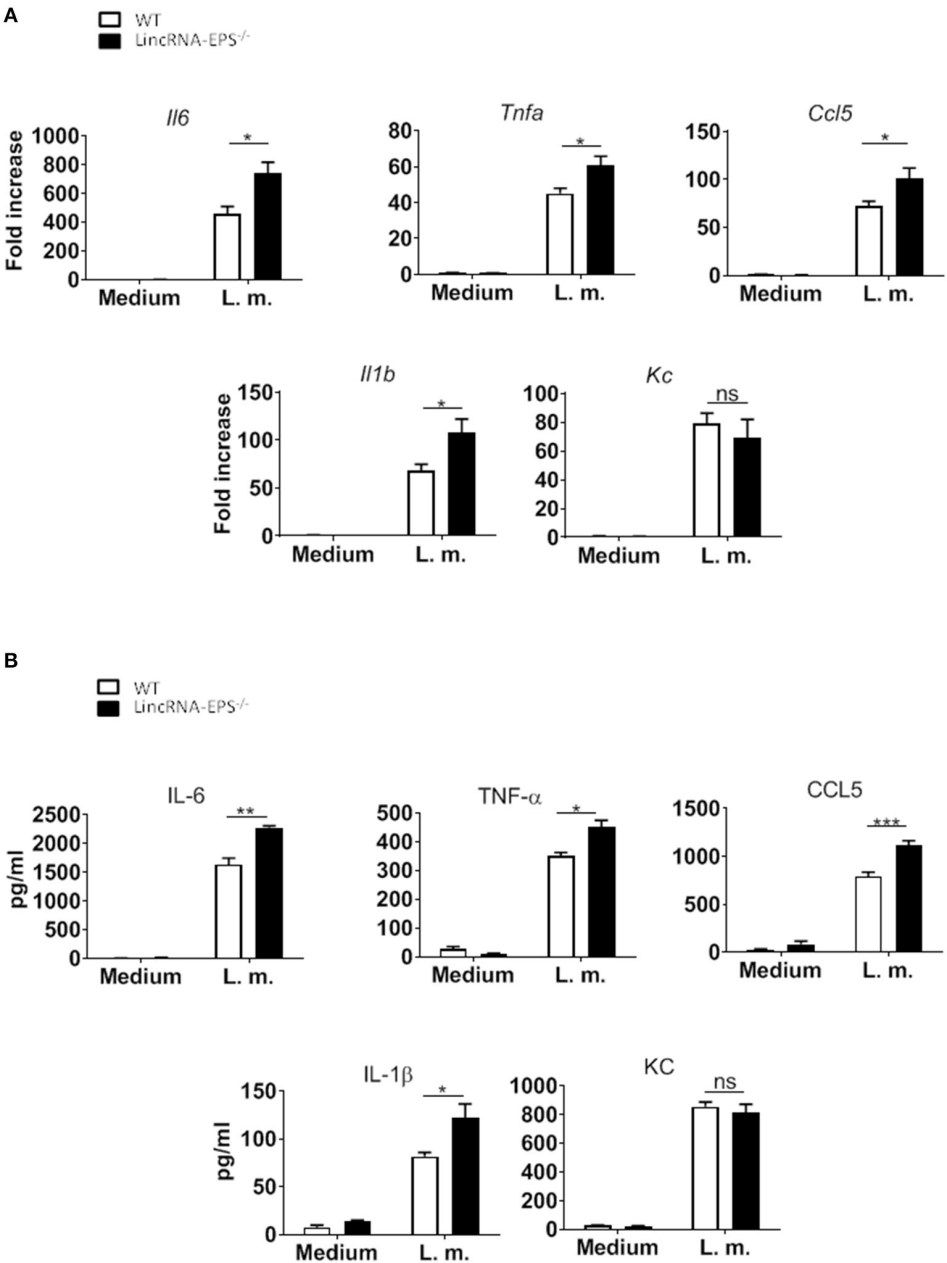
LincRNA-EPS restrains *L. monocytogenes*-induced immune gene expression in BMDMs. **(A)** mRNA levels of cytokine and chemokine genes in WT and lincRNA-EPS^−/−^ BMDMs after 3 h of *L. monocytogenes* infection (MOI of 1). **(B)** Protein levels of cytokines and chemokine in WT and lincRNA-EPS^−/−^ BMDMs after 6 h of *L. monocytogenes* infection (MOI of 1). An unpaired, two-tailed Student's *t*-test was performed to determine statistical significance. Data are shown as mean ± SEM of at least two independent experiments. ***p = 0.0006; **p = 0.001; *^1^*p* = 0.03; *^2^*p* = 0.02; *^3^*p* = 0.05; *^4^*p* = 0.03; *^5^*p* = 0.02; *^6^*p* = 0.02; ns, not significant.

A pervious study showed that the expression of lincRNA-EPS is downregulated in WT BMDMs infected with *L. monocytogenes* (Atianand et al., 2016). Furthermore, lincRNA-EPS is a negative regulator of inflammatory response in mouse macrophages exposed to LPS (Atianand et al., 2016). As infection by *L. monocytogenes* is initially controlled by macrophages (Wing and Remington, 1977), we wondered whether lincRNA-EPS might play a role in macrophages during this bacterial infection. For this purpose, BMDMs isolated from WT and lincRNA-EPS^−/−^ mice were infected with *L. monocytogenes* and we measured the expression of proinflammatory cytokines and chemokines at both mRNA and protein levels. We found that *L. monocytogenes*-induced expression of *Il6, Tnfa, Il1b* and *Ccl5* was significantly upregulated in lincRNA-EPS^−/−^ BMDMs compared to control cells (Figure 1A); whereas no difference was observed in KC expression. These results were further confirmed at the protein level (Figure 1B). Taken together these data indicate that lincRNA-EPS acts as restrainer of *L. monocytogenes-*induced immune genes expression in mouse macrophages.

### LincRNA-EPS Controls *L. monocytogenes*-Induced Immune Gene Expression in Mouse Dendritic Cells

**Figure 2:**
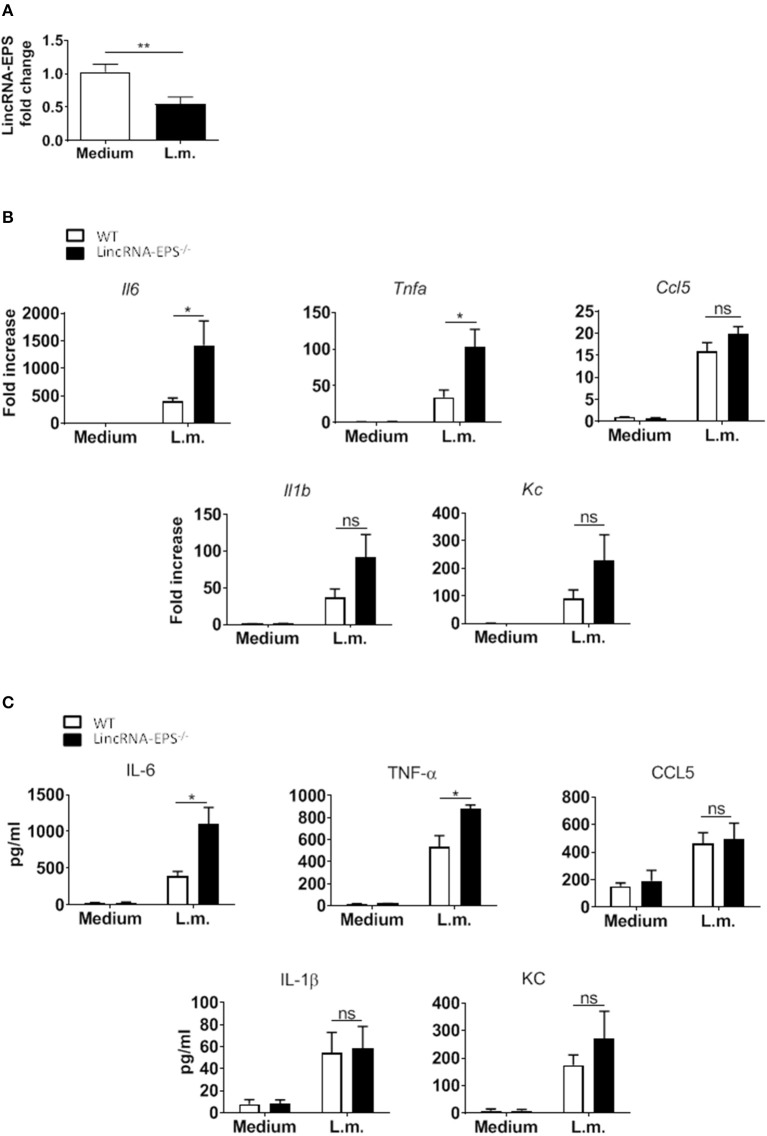
LincRNA-EPS restrains *L. monocytogenes*-induced immune gene expression in BMDCs. **(A)** LincRNA-EPS mRNA level in WT BMDCs after 6 h of *L. monocytogenes* infection (MOI of 1). **(B)** mRNA levels of cytokine and chemokine genes in WT and lincRNA-EPS^−/−^ BMDCs after 6 h of *L. monocytogenes* infection (MOI of 1). **(C)** Protein levels of cytokines and chemokines in WT and lincRNA-EPS^−/−^ BMDCs after 6 h of *Listeria* infection (MOI of 1). Unpaired, two-tailed Student's *t*-test was performed to determine statistical significance. Data are shown as mean ± SEM of at least two independent experiments. **p = 0.009; *^1^*p* = 0.05; *^2^*p* = 0.01; *^3^*p* = 0.03; *^4^*p* = 0.02; ns, not significant.

Next, we hypothesized that following *L. monocytogenes* infection lincRNA-EPS might have a role in innate immune cell types other than macrophages. Dendritic cells are innate immune cells well known to be involved in *L. monocytogenes* infection (Serbina et al., 2003; Tam and Wick, 2004). As observed in macrophages, DCs also downregulate lincRNA-EPS expression following *L. monocytogenes* infection (Figure 2A). We further measured the levels of immune related cytokines and chemokines produced by WT and lincRNA-EPS^−/−^ BMDCs. We found that compared to WT cells, IL-6, and TNF-α were significantly up regulated in lincRNA-EPS^−/−^ BMDCs at both mRNA (Figure 2B) and protein levels (Figure 2C); on the contrary, the expression of IL-1β, CCL5, and KC was not altered in lincRNA-EPS^−/−^ cells. Collectively, these results demonstrate that lincRNA-EPS expression is negatively regulated in response to *L. monocytogenes* infection in mouse dendritic cells and lincRNA-EPS downregulation leads to the upregulation of proinflammatory cytokines, such as IL-6 and TNF-α.

### LincRNA-EPS Promotes *L. monocytogenes* Replication *in vitro* and *in vivo*

**Figure 3:**
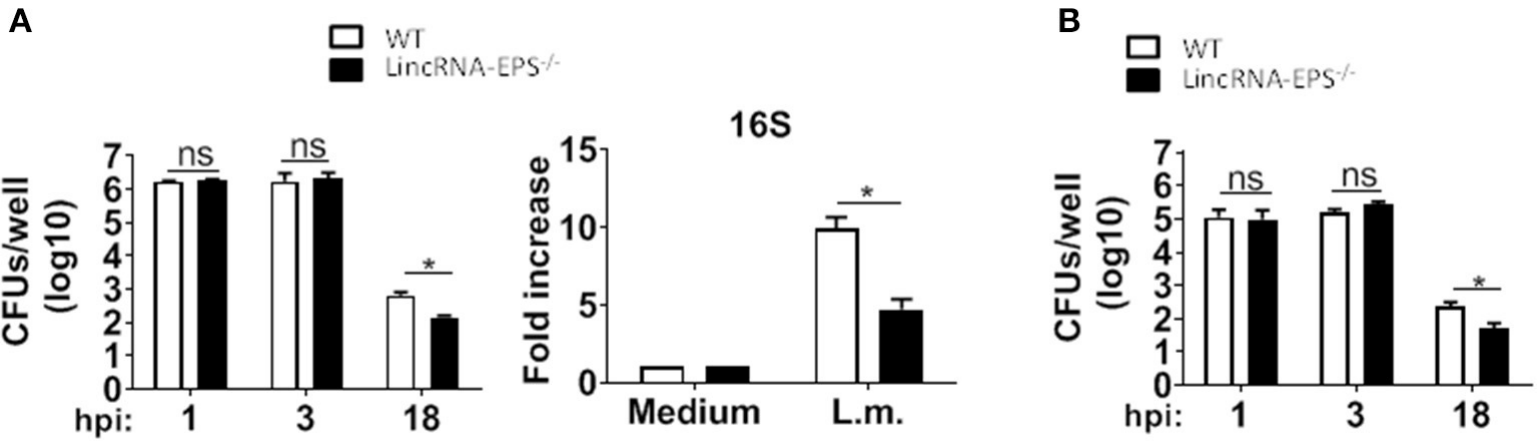
LincRNA-EPS promotes *L. monocytogenes* replication *in vitro*. **(A)** CFU counts in WT and lincRNA-EPS^−/−^ BMDMs after 18 h of *Listeria* infection (MOI of 1) (left) and mRNA levels of bacterial 16S in WT and lincRNA-EPS^−/−^ BMDMs after 6 h of *L. monocytogenes* infection (MOI of 1) (right). **(B)** CFU counts in WT and lincRNA-EPS^−/−^ BMDCs after 18 h of *L*. monocytogenes infection (MOI of 1). Unpaired, two-tailed Student's *t*-test was performed to determine statistical significance. Data are shown as mean ± SEM of at least two independent experiments. *^1^*p* = 0.01; *^2^*p* = 0.04; *^3^*p* = 0.04; ns, not significant.

**Figure 4:**
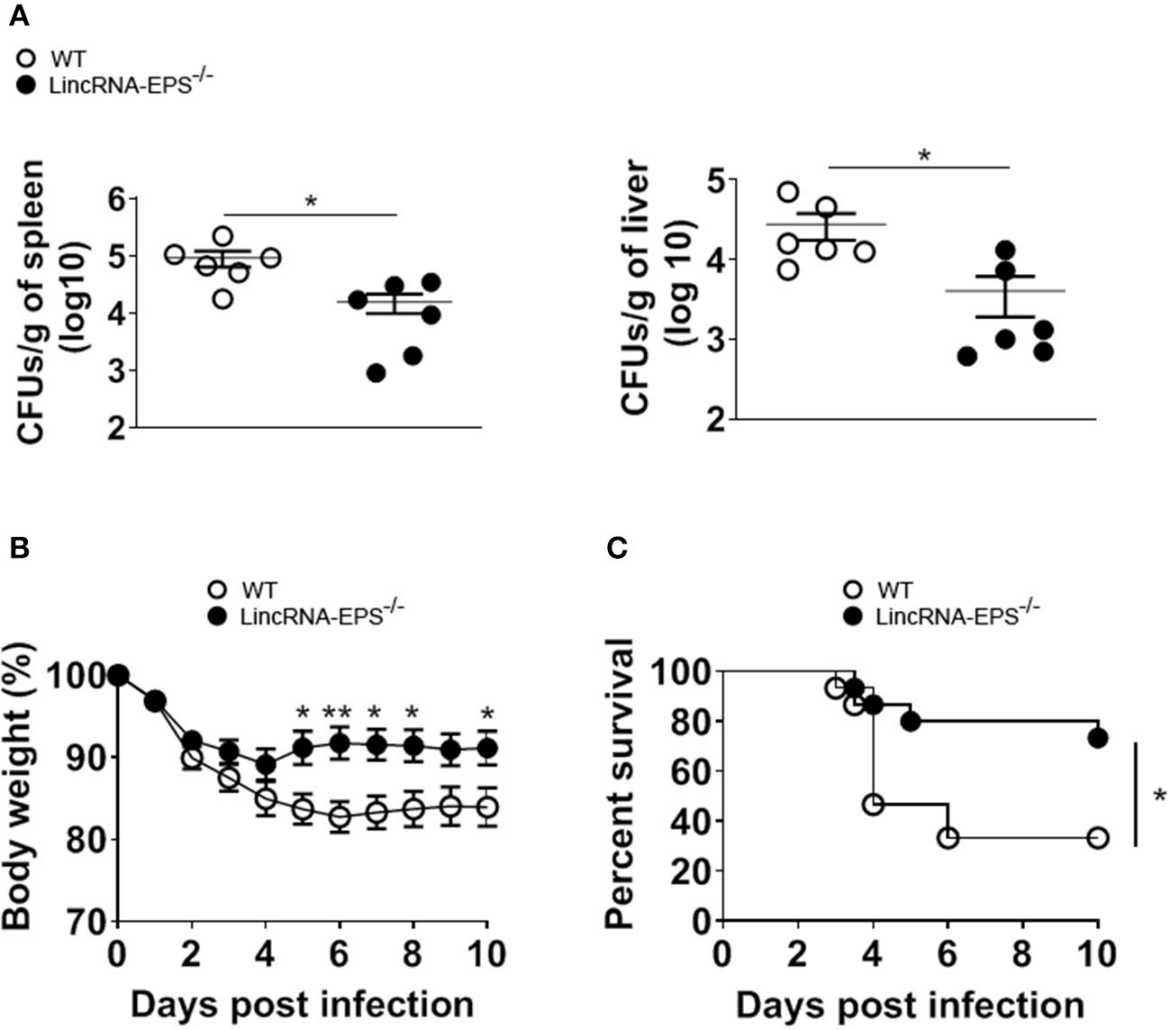
LincRNA-EPS promotes *L. monocytogenes* replication *in vivo*. **(A)** CFU count in 1 g of spleen (left) and liver (right) of WT and lincRNA-EPS^−/−^ mice after 72 h of *L. monocytogenes* infection (10^5^ CFUs/mouse). **(B)** Weight loss in WT and lincRNA-EPS mice monitored for 10 days after i.p. *Listeria* monocytogenes infection (5 × 10^5^ CFUs/mouse). **(C)** Survival rate in WT and lincRNA-EPS^−/−^ mice monitored for 10 days after i.p. *L. monocytogenes* infection (5 × 10^5^ CFUs/mouse). In **(A)** unpaired, two-tailed Student's *t*-test was performed to determine statistical significance. In **(B)** two-way ANOVA with the Bonferroni post-hoc test was performed to determine statistical significance. In **(C)** a Log-rank (Mantel-Cox) test was performed to determine statistical significance. Data are shown as mean ± SEM of at least two independent experiments. **p = 0.003; *^1^*p* = 0.03; *^2^*p* = 0.05; *^3^*p* = 0.03; *^4^*p* = 0.01; *^5^*p* = 0.02; *^6^*p* = 0.04.

Next, we examined whether the *L. monocytogenes*-induced lincRNA-EPS downregulation might affect the intracellular bacterial replication in mouse macrophages and dendritic cells. Infection of WT and lincRNA-EPS^−/−^ BMDMs or BMDCs with *L. monocytogenes* resulted in a comparable number of bacterial colony forming units (CFUs) immediately after infection [between 1 and 3 h post infection (hpi)] (Figures 3A,B). However, compared with WT cells, significantly lower CFUs were recovered from lincRNA-EPS^−/−^ BMDMs and BMDCs at 18 hpi with *L. monocytogenes* (Figures 3A,B). Furthermore, the bacterial 16S ribosomal mRNA measured at 6 hpi by qPCR was significantly lower in lincRNA-EPS^−/−^ BMDMs compared to WT cells (Figure 3A). LincRNA-EPS was shown to restrain inflammation in mice exposed to LPS, with a significantly higher mortality rate in lincRNA-EPS^−/−^ mice (Atianand et al., 2016). However, the role of lincRNA-EPS during bacterial infection *in vivo* remains unknown. We noticed that lincRNA-EPS expression is significantly downregulated in the spleen following *L. monocytogenes* infection (Supplementary Figure 1A). Furthermore, after *L. monocytogenes* infection of WT and lincRNA-EPS^−/−^ mice, we monitored bacterial clearance and cytokine responses. As shown in Figure 4A, significantly lower CFUs were detected at 48 hpi in the spleen and liver of *L. monocytogenes*-infected lincRNA-EPS^−/−^ mice compared to WT mice, suggesting that lincRNA-EPS enhances bacterial replication *in vivo*. However, none of the analyzed cytokines showed altered expression in the infected lincRNA-EPS^−/−^ mice compared to WT (Supplementary Figures 1B,C). Furthermore, we infected WT and lincRNA-EPS^−/−^ mice with a lethal dose of *L. monocytogenes* and we monitored them for weight loss and survival. Strikingly, lincRNA-EPS^−/−^ mice lost significantly less body weight than WT mice (Figure 4B) and consistent with this result, lincRNA-EPS^−/−^ mice were significantly less susceptible to *L. monocytogenes* infection. In fact, 70% of WT mice succumbed to death, whereas only 25% of the lincRNA-EPS^−/−^ mice died (Figure 4C). Collectively, these results indicate that during *L. monocytogenes* infection, lincRNA-EPS promotes bacterial survival in both mouse macrophages and dendritic cells and has also a crucial role in bacterial clearance and host survival *in vivo*.

### LincRNA-EPS Inhibits *iNos* Expression and NO Production

**Figure 5:**
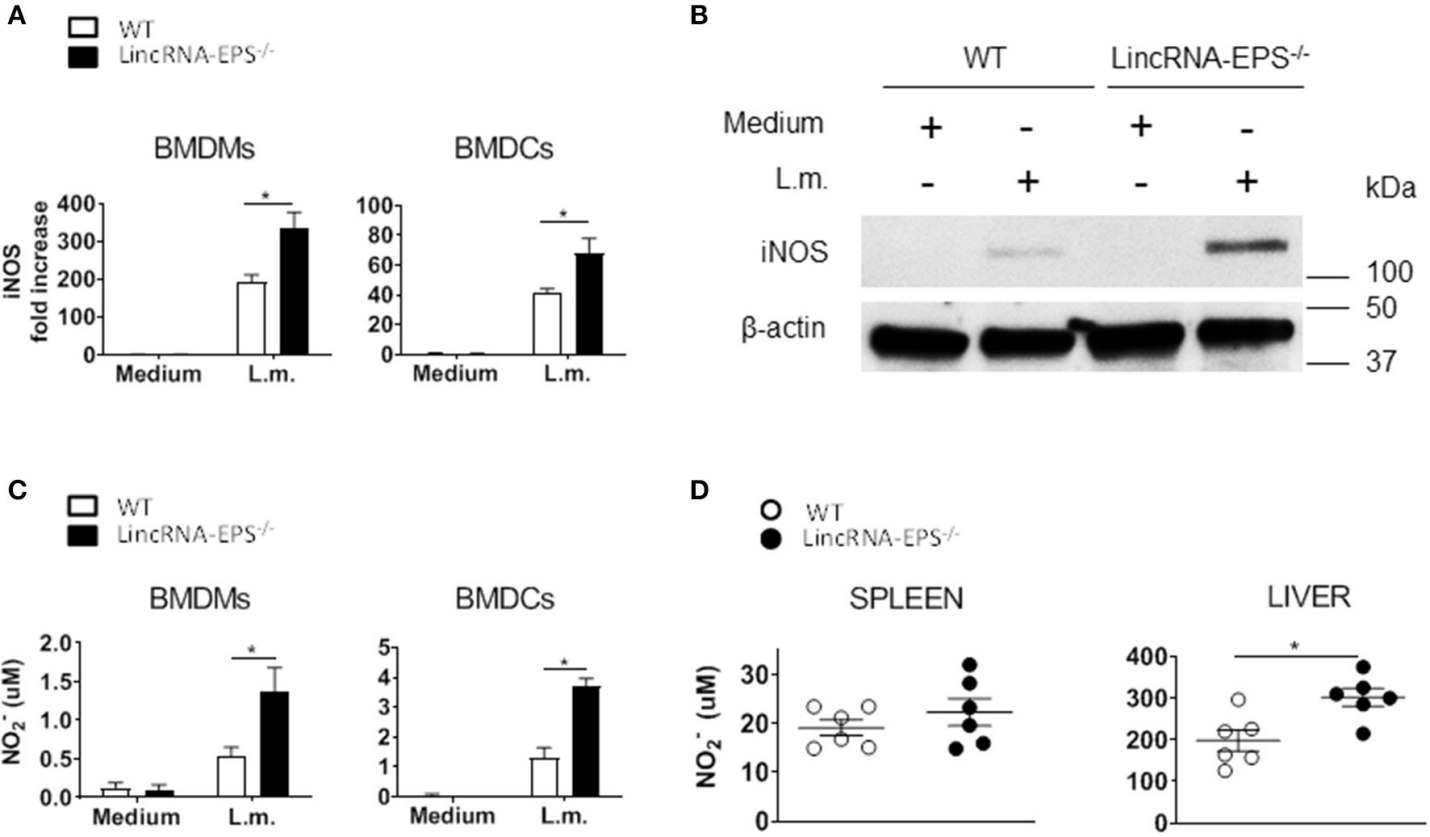
LincRNA-EPS restrains *L. monocytogenes*-induced *iNos* expression and NO production. **(A)** mRNA levels of *iNos* in WT and lincRNA-EPS^−/−^ BMDMs (left) and BMDCs (right) after *L. monocytogenes* infection (MOI of 1). **(B)** Immunoblots of iNOS and β-actin in lysates of BMDMs untreated or stimulated with *L. monocytogenes* (MOI of 10) for 6 h. **(C)** NO production in WT and lincRNA-EPS^−/−^ BMDMs (left) and BMDCs (right) after 48 h of *L*. *monocytogenes* infection (MOI of 1). **(D)** NO production in spleen (left) and liver (right) of WT and lincRNA-EPS^−/−^ mice after 48 h of *L. monocytogenes* infection (10^5^ CFUs/mouse). Unpaired, two-tailed Student's *t*-test was performed to determine statistical significance. Data are shown as mean ± SEM of at least two independent experiments. *^1^*p* = 0.03; *^2^*p* = 0.03; *^3^*p* = 0.01; *^4^*p* = 0.01; *^5^*p* = 0.01.

Enhanced bacterial clearance *in vitro* and *in vivo* in the absence of lincRNA-EPS suggested that lincRNA-EPS might be suppressing cellular antimicrobial mechanisms. Several studies showed that induced iNOS and nitric oxide (NO) are critical antimicrobic mediator in host defense against *L. monocytogenes* (Boockvar et al., 1994; Macmicking et al., 1995; Serbina et al., 2003). Thus, we hypothesized that during *L. monocytogenes* infection, lincRNA-EPS might be crucial in controlling iNOS expression and the consequent NO production. Toward this end, we exposed BMDMs and BMDCs to *L. monocytogenes* and analyzed *iNos* expression by qPCR at 3 and 6 hpi, respectively. We found that BMDMs and BMDCs from lincRNA-EPS^−/−^ mice express significantly higher levels of iNOS than WT at both mRNA and protein levels (Figures 5A,B). Consistently, lincRNA-EPS^−/−^ macrophages and dendritic cells produce significantly higher amounts of NO compared to control cells (Figure 5C). We further tested the effect of lincRNA-EPS on NO production *in vivo*. As shown in Figure 5D, NO production measured in liver of lincRNA-EPS^−/−^ mice at 48 hpi was significantly higher than WT mice, and a similar trend, albeit of lower magnitude, was observed in the spleen. Taken together these results demonstrate that lincRNA-EPS controls *iNos* expression and the production of NO during *L. monocytogenes* infection, which could impair bacterial clearance.

## Discussion

LncRNAs are emerging as regulators of inflammatory responses of immune cells and host-pathogen interactions. LincRNA-EPS is a nuclear intergenic long non-coding RNA showed to act as a transcriptional brake to restrain inflammation (Atianand et al., 2016). Furthermore, lincRNA-EPS is downregulated in mouse macrophages after infection with the gram-positive bacterium *Listeria monocytogenes* (Atianand et al., 2016). However, the role of lincRNA-EPS in host responses to bacterial infections remained unknown.

In this paper we showed that macrophages lacking lincRNA-EPS exhibit increased expression of inflammatory cytokines such as TNF-α, IL-6, IL1-β, and CCL5 upon infection with *L. monocytogenes*. Consistently, compared to WT cells, lincRNA-EPS-deficient dendritic cells infected with *L. monocytogenes* showed upregulated expression of TNF-α and IL-6; whereas IL1-β and CCL5 were not differentially expressed. Thus, lincRNA-EPS seems to exert a stronger effect in BMDMs, which are likely to be more sensitive to lincRNA-EPS-dependent molecular events than are BMDCs. Another possibility might be that the constitutive expression of lincRNA-EPS is higher in BMDMs, where it exerts a more robust transcriptional control.

Furthermore, we showed that the expression of lincRNA-EPS is strongly downregulated in BMDCs during *L. monocytogenes* infection, as it was shown in BMDMs (Atianand et al., 2016). Although it is still not known what drives lincRNA-EPS downregulation in response to *L. monocytogenes* infection, it is likely that the host responds to *L. monocytogenes* inducing the expression of one or more molecules that might be able to promote the downregulation of lincRNA-EPS, which would be a result of the host molecular mechanism of defense. Alternatively, a *Listeria*-derived molecule might directly or indirectly induce the downregulation of lincRNA-EPS. Furthermore, Atianand and al. showed that the TLR-dependent suppression of lincRNA-EPS was dependent on MyD88 and TRIF (Atianand et al., 2016), thus these two molecules might be also critical in modulating the expression of lincRNA-EPS during *L. monocytogenes* infection.

Compared with WT cells, lincRNA-EPS-deficient BMDMs and BMDCs showed equal infectivity at the start of infection, indicating that the increased cytokine responses cannot be explained by higher microbial burdens. Of note, at late time points post-infection, both BMDMs and BMDCs lacking lincRNA-EPS harbored lower bacterial loads, suggesting enhanced bacterial clearance. These findings were supported by a higher production of iNOS and nitrates in lincRNA-EPS^−/−^ cells compared to controls. To uncover the role of lincRNA-EPS in eliciting immune responses during infections with intracellular bacteria *in vivo*, we analyzed cytokine expression in WT and lincRNA-EPS^−/−^ mice after systemic infection with *L. monocytogenes*. Although the loss of lincRNA-EPS did not alter the cytokine production in spleen and liver of mice infected with *L. monocytogenes*, NO production is higher in lincRNA-EPS-deficient mice compared to WT. The lack of differences in cytokine levels *in vivo* between WT and lincRNA-EPS^−/−^ mice might be explained by the fact that lincRNA-EPS may be mainly expressed and has a prominent effect in splenic and liver macrophages and dendritic cells; thus, any possible differences in these two populations would be masked by other cells types, where lincRNA-EPS might not play a role. An alternate possibility is that unlike its important role in iNOS expression and bacterial clearance, lincRNA-EPS's role in cytokine responses might be redundant *in vivo*. Consistent with the higher NO production, bacterial CFUs in spleen and liver following i.p. infection with *L. monocytogenes* were significantly reduced in lincRNA-EPS^−/−^ mice compared to WT. Published studies provide strong evidence that inhibition of iNOS expression results in increased susceptibility to listeriosis (Macmicking et al., 1995; Serbina et al., 2003); furthermore, NO is one of the major bactericidal mediators involved in host defense against *L. monocytogenes* (Boockvar et al., 1994). Therefore, it is possible that the enhanced bacterial clearance and host survival observed in lincRNA-EPS^−/−^ mice could be mediated by the elevated NO production.

At a steady state, lincRNA-EPS has been shown to maintain a repressed chromatin state at immune related genes, inhibiting their expression (Atianand et al., 2016). Furthermore, lincRNA-EPS interacts with the ribonucleoprotein hnRNPL (Atianand et al., 2016), known to control transcriptional regulation of gene expression (Huang et al., 2012; Giraud et al., 2014; Li et al., 2014). Thus, it is likely that during *L. monocytogenes* infection, downregulation of lincRNA-EPS leads to an increased production of proinflammatory cytokines and iNOS in splenic and liver macrophages and dendritic cells, followed by a general enhancement of NO production *in vivo*. This results in a more efficient immune response and bacterial clearance when lincRNA-EPS is completely absent. However, further studies are necessary in order to better elucidate the molecular mechanism by which lincRNA-EPS restrains iNOS during *L. monocytogenes* infection. In summary, our study reveals a critical role for lincRNA-EPS in anti-bacterial host defense. These findings further improve our understanding of the role of lncRNAs in host-pathogen interactions and in the regulation of immune responses during infection.

## Data Availability Statement

All datasets generated for this study are included in the article/Supplementary Material.

## Ethics Statement

The animal study was reviewed and approved by Institutional Animal Care and Use Committee (IACUC).

## Author Contributions

FA conducted the experiments with *L. monocytogenes in vitro* and *in vivo*, analyzed the results, and wrote the manuscript. KF provided LincRNA-EPS^−/−^ mice. AM, AV, and VR conceptualized the research and finalized the manuscript.

### Conflict of Interest

The authors declare that the research was conducted in the absence of any commercial or financial relationships that could be construed as a potential conflict of interest.
