# Supplementary material for: Long Non-coding RNA LincRNA-EPS Inhibits Host Defense Against Listeria monocytogenes Infection
Source: Front Cell Infect Microbiol. 2020 Jan 22;9:481. doi: 10.3389/fcimb.2019.00481 (PMC6987077; doi:10.3389/fcimb.2019.00481)
Supplement: Supplementary file 1 [file Data_Sheet_1.PDF]

Supplementary Material

1.1 Supplementary Figures

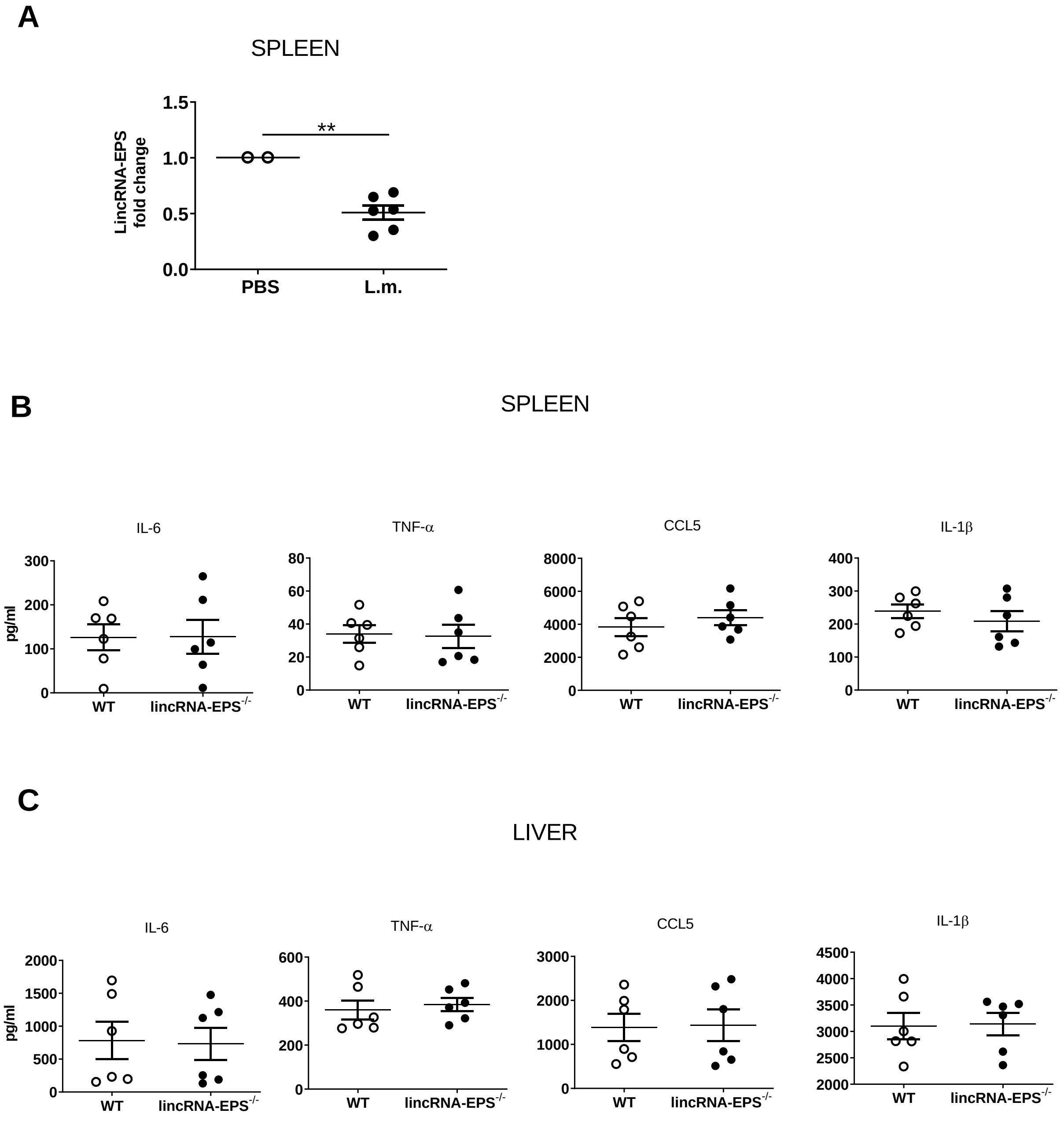

Supplementary Figure S1. LincRNA-EPS does not alter *L. monocytogenes*-induced immune gene expression *in vivo*. (A) LincRNA-EPS mRNA in spleen of WT mice after 48h of i.p. *L.* monocytogenes infection (10^5 CFUs/mouse). Protein levels of cytokines in the

spleen (B) and liver (C) of WT and lincRNA-EPS$^{-/-}$ mice after 48h of i.p. *L. monocytogenes* infection (10^5 CFUs/mouse). Data are shown as mean ±SEM of two independent experiments. **$p<0.01$; ns, not significant.
